# Stress and Traditional Support: The Role of Orphans’ and Vulnerable Children’s Primary Caregivers in Rural Ethiopia

**DOI:** 10.3390/children12010096

**Published:** 2025-01-16

**Authors:** Aweke Tadesse, Kenan Li, Jesse Helton

**Affiliations:** 1 School of Social Work, Saint Louis University, St. Louis, MO 63103, USA; 2 College of Public Health and Social Justice, Saint Louis University, St. Louis, MO 63103, USA; kenan.li@slu.edu (K.L.); jesse.helton@slu.edu (J.H.)

**Keywords:** perceived stress, primary caregivers, well-being, traditional support, spirituality, community cohesion

## Abstract

Background/Objectives: Orphans’ and Vulnerable Children’s (OVC) primary caregivers (PCGs) in Ethiopia live with multiple social and emotional problems stemming from extreme poverty, war, environmental disasters, and the HIV pandemic. Family and community supports are strained, leaving OVC’s PCGs dependent on inconsistent humanitarian aid. This aid is typically focused on OVCs and does not address PCG well-being. This study aimed to assess PCG well-being and explore their perceived stress and the traditional family and community coping mechanisms. Method: Participants from the Oromia region of Ethiopia were recruited, and a mixed-methods design was employed. Six cases were included using a criterion sampling technique. An explanatory data analysis technique was used, and data were triangulated from interviews, focus group discussions, archival information, and surveys. A 36-item Well-being Measuring Tool (WMT) Likert scale was employed to measure well-being domains. Result: The results indicated that none of the PCGs reported the desirable and average summative well-being domain mean scores [25 and 23]. Among the ten well-being domains, only “spirituality” and “community cohesion” were above the average score [2.3]. The “economy” domain had the lowest score, indicating a critical deficiency [1.3]. A lack of community support could exasperate perceived stress, and well-being deficiencies were linked. Conclusions: Lack of family and community support could exacerbate feeling overlooked, unwelcome, and lonely. A policy that promotes a supportive community environment and safeguards the most vulnerable OVCs and their PCGs should be in place. This study recommends further rigorous research examining the well-being determinants of OVCs’ PCGs in Ethiopia and the role of traditional support systems in rural settings.

## 1. Introduction

Caregiver stress is often heightened in rural and low-income settings due to limited access to resources and support systems, which are crucial for managing parenting demands. In sub-Saharan Africa, factors such as household income and educational level significantly influence parental stress, with higher socioeconomic status serving as a buffer, particularly in regions where systemic challenges amplify the burden of caregiving [1]. Orphans’ and Vulnerable Children’s (OVCs’) primary caregivers (PCG) in Africa overwhelmingly bear the stress of not only their own lives but also of their children’s lives. Operationally, PCGs are mostly the heads of the household, including biological parents, grandparents, relatives, neighbors, and other guardians with non-blood relationships who provide care and support for OVCs [2,3]. PCGs are placed in a position to care for these children due to the loss of life from the HIV-AIDS pandemic, poverty, war, environmental disasters, and other humanitarian stressors. Yet, there are no centralized supportive strategies from the local government, reinforcing policies, or financial support systems that could ease this socioeconomic stress and instability [4,5]. Parental loss is devastating and has a long-term effect on children’s lives. Research shows that OVCs’ overall well-being is linked to the PCG’s quality of life and mental health, which could also impact their caring role and coping skills [6,7]. Operationally, well-being refers to the overall condition of PCGs and their OVC’s state of health, happiness, and comfort, including socioeconomic, mental, emotional, and physical functioning [8]. OVCs from the most vulnerable family and home environments commonly have stressful peer involvement, low peer acceptance, and a high probability of mental disorders [5].

Studies from sub-Saharan Africa (SSA) and others suggest the need for a sustainable support approach that could enhance OVCs’ PCGs’ economic stability, legal protection, social cohesion, educational attainment, health, and psychological care [9,10,11,12]. For example, among these studies, Ariyo (2019) and Nelson (2010) reported that household socioeconomic conditions were the determining factors for SSA’s child well-being [9,12]. These studies also suggested the need for support strategies that could help strengthen low-income families’ economic and social capacity through institutional support and local community care and support mechanisms such as kinship care. For example, similar studies from four countries (Ethiopia, Kenya, Cambodia, and India) found that support from religious entities has a positive role in sustaining OVCs’ caregivers’ coping mechanisms as they carry their own and directly share their OVC challenges [13]. Research in China found that direct material support for families in hardship improved child development care by reinforcing helpful caregiver practice toward children’s socio-emotional development [14]. A study from Swaziland showed that OVC caregivers who had access to community financial inclusion opportunities, such as savings and access to loans, were allowed to increase their earnings, which could be associated with positive health impacts on themselves and their OVCs [15]. Other empirical studies from North Vietnam and the USA have highlighted a significant relationship between the financial constraints faced by grandparent caregivers and the well-being challenges, including physical and emotional difficulties, experienced by the PCGs [16,17]. This study explored the well-being condition of OVCs’ PCGs and the role of traditional support systems.

### 1.1. Background: Humanitarian Condition in Ethiopia

Ethiopia is one of the poorest and most populous countries in Africa, with a total population of over 120 million, 13 million of whom are under five years old, 6% (5.5 million) of vulnerable OVCs, and a 48 per 1000 infant mortality rate [18,19]. Multiple factors exacerbate the vulnerability of OVCs and their PCGs in Ethiopia [20]. These factors include HIV/AIDS, poverty, war, violence, economic inequality and a lack of equity and protection, corruption, and climate-related disasters. The country is home to tragic ethnic conflict, internal displacement, external migration, war, and hunger. For example, while the COVID pandemic has severely affected the country’s development initiatives and deteriorated individual and household livelihoods since 2020, simultaneously, a tragic ethnic conflict between the federal government and rebel groups in the regional states of Oromia and Tigray was active until the beginning of 2023 and resulted in millions of internally displaced people (IDPs), starvation, and the loss of beloved ones. For example, the country’s nutritional deficiencies accounted for over 45% of child deaths [21]. Continuous organized rebel attacks and insecurity exist nationwide [22]. An International Displacement Monitoring Center (IDMC) report ranked Ethiopia as having the highest number of IDPs worldwide, with an estimated total of over two million by the end of 2020 [22,23].

Ethiopia had the second-highest OVC population worldwide. In recent years (i.e., 2019–2023), climate changes, such as drought and hunger, have severely affected the East African country’s humanitarian livelihood and stability. For example, in 2022–2023, an active and critical food shortage and drought affected over 20 million Ethiopians and involved several international humanitarian organizations in relief and crisis management [24]. Some studies have categorized Ethiopia as “fragile” as the country continuously remained in economic disaster, with increased unemployment, internal displacement, migration, and inflation rates [25,26]. For example, according to Destaa’s study, between 2010 and 2016, there was a consistent GDP growth of 9.9 in the country; however, it dropped from 8.36 in 2019 to 6.06 in 2020. Similarly, the unemployment rate increased from 19% to 21.6% between the years 2019 and 2021 [27].

The condition of a PCG’s well-being, the availability of basic needs, and a safe and healthy home environment are important factors in determining the quality of life and the quality of a caregiving role for OVCs. Parental loss, grief, and unmet basic needs affect the holistic growth of OVCs, including their psychological functioning, social and cognitive skills, emotions, and behaviors [28]. Overwhelmingly, the COVID-19 pandemic exacerbated the poor livelihood of OVCs and their PVGs, more tragically, in low-income countries like Ethiopia. The adverse effect of the pandemic is not only worsening the well-being of OVCs and their vulnerable PCGs but is also a factor in increasing the number of OVCs worldwide and leaving many children with a single parent or no biological parent. According to UNICEF, there are more than 153 million orphans worldwide; 14 million have lost both parents, and 9 million are in SSA [29]. In this study, according to UNICEF’s report, over 1,134,000 children lost their PCGs, including biological parents.

### 1.2. Supportive Mechanisms for OVCs’ Primary Caregivers in Ethiopia

Family, friends, and neighborhood support impact individual and community well-being, likely through mechanisms that connect vulnerable individuals with groups of supportive people [29,30]. The family, especially in rural Ethiopia like the Ziway area, refers to a patriarchal type of extended family where the father and eldest son are viewed as the heads of the nuclear family who work and live together [31]. Studies have established a link between social support, QOL, mental health, loneliness, and satisfaction [30,32]. In life, community cohesion and neighborhood connectedness likely impact well-being and health outcomes [33]. Studies showed that individual affiliative environments, such as connectedness and perceived social acceptance, were correlated with a lower probability of isolation, loneliness, and depression and lower life satisfaction [34,35].

Child nurturing is an enormous responsibility that requires emotional commitment, time, and financial investment. Low-income OVCs’ PCGs are burdened and strongly challenged in addressing their own basic needs and those of the OVCs, despite their commitment and striving to cope with deteriorating conditions of well-being at a later age in life. Seeing unmet children’s needs daily; being hungry, without proper shelter, clothing, and healthcare; being isolated and discriminated against; and dropping out of school are stressors against PCG well-being [36]. Family and community support systems and institutional interventions are commonly used to assist vulnerable OVC caregivers. However, the existing care and support remain unsatisfactory due to multiple factors that have increased the number of OVCs, the demand for living and unstable socioeconomic conditions, and the deterioration of PCGs’ health conditions in Ethiopia [13]. Commonly, the focus of many humanitarian interventions gives extensive weight to the immediate losses of the OVCs but places less emphasis on PCG well-being needs. Apart from institutional care, such as orphanages, non-institutional intervention is also provided by many NGOs in Ethiopia, including Food for the Hungry, World Vision, Compassion International, Plan Ethiopia, and others, including sponsorship programs through churches that assist with child education material, medical support, clothing, and food in selected areas through coordination with the government [37]. These care and support provisions have enabled many OVCs to attend their schools and, to some extent, relieved their PCGs. Yet, due to a lack of sustainability models in the approach, OVCs’ PGCs were still stressed and worried about the future as they constantly felt dependent.

This study explored the conditions of families and neighborhood supportive systems connected to OVCs’ PCGs’ well-being and their experience in the caring role. The study was guided by two questions: What major factors affect OVCs’ PCGs’ well-being and are linked with their caring role? What supportive systems assisted OVCs’ PGCs’ role in coping with their challenges? This study’s main goal was to explore the major factors connected with PGCs’ caring role and well-being that could implicitly and explicitly affect their quality of living. In particular, the study explored factors connected with PCGs’ perceived stress and the family and community support systems that assisted PCGs in coping and being resilient in their caring roles.

## 2. Research Methodology

### 2.1. Design

This case study applied a purposive sampling method. Data were collected using a mixed-methods approach, quantitative and qualitative tools, in a sequential pattern for the purpose of triangulation. First, a pilot study was conducted, and a few semi-structured questions were revised. Second, structured questionnaires assessed PCGs’ well-being domains using a 36-item Likert scale. Third, an in-depth investigation was conducted using semi-structured questions with six PCGs and key informant groups filed by community promoters and program managers. The qualitative data collection method used face-to-face interviews, focused group discussions with key informants, individual key informant informal interviews, and case file reviews. Besides the six targeted cases, information was collected both quantitatively and qualitatively, from key informant participants through group discussion (*n* = 11) and individual interviews (*n* = 6). The participants included FHE program managers, officers, and field promoters who had roles closely working with the study participants.

Scholars indicated that a case study approach allows a variety of investigation methods (i.e., both qualitative and quantitative) in collecting data and triangulating findings intriguingly and gives researchers room for some flexibility [38]. According to Yin and Creswell, for a real-life and contemporary phenomenon in a real setting interested in exploring “how” and “why” questions in depth, a case study is a preferred approach [39,40]. The study used a Well-being Measuring Tool (WMT) widely used in Africa to measure ten key well-being domains in community intervention settings, including food and nutrition; shelter; protection; family spirituality; mental health; education; economy; and community cohesion. For example, Catholic Relief Services (CRS) have used WMTs in SSA OVC programs that have been operational, including Ethiopia, Kenya, and Rwanda. The tool is useful for field practitioners manually monitoring individual conditions of well-being domains and taking intervention actions accordingly and for a macro well-being survey analyzed using a computer program [41].

### 2.2. Study Population and Site

The study area is in Ethiopia Rift Valley, Oromia region, East Showa Zone, Ziway district. Ziway is an area town with an estimated total population of 49.4 thousand. The district is characterized by irregular rainfall (annual rainfall of 760 mm) and drought-prone areas. The altitude ranges from 1500 to 2500 m above sea level with an estimated average temperature of 200 centigrade. Multiple private flower farms have attracted thousands of low-skilled and daily laborers to the town. Ziway is home to many ethnic and religious groups, which has contributed to serious resource-based ethnic tensions and conflicts. The overflow of daily laborers and high unemployment has also exposed the area to HIV infection, insecurity, and other social and economic problems. These critical humanitarian issues opened a door for Food for the Hungry Ethiopia (FHE) to perform community intervention, including care and support for OVCs and their caregivers, ultimately focusing on the most vulnerable and poor households.. FHE is a community-committed non-governmental international organization (NGO) closely working with local community leaders, families, and churches. Community-level staff (promoters) were key in the implementation strategy and followed the PCG’s OVC school performance and overall well-being. The study cases were initially selected by the joint criteria and agreement of FHE and local leaders and defined as “the most vulnerable OVC households/caregivers”.

### 2.3. Researcher

The data were collected and analyzed by the first author, an Ethiopian who studied his undergraduate education in Ethiopia. Currently (2023), he is a Social Work Ph.D. candidate and a graduate research assistant at a large, private Catholic university in the Midwest of the USA. He also earned a Ph.D. in Holistic Child Development (HCD) in the Philippines and teaches graduate social work classes at his university. Over a decade, he has led development intervention programs in Mozambique and Ethiopia, including income generation (saving groups), Food for Assets (FFA), Child Development, Disaster Mitigation, WASH, and Health and Nutrition. He has had the chance to visit hundreds of OVC households, work with their caregivers, and facilitate special assistance to Vulnerable OVC PCGS. Between 2007 and 2018, he designed and conducted multiple development capacity-building training and monitoring activities for community development professionals and social service leaders in Ethiopia and Mozambique.

### 2.4. Selection Criteria

The selection of OVCs’ PCGs used the following criteria: the PCG was identified as one of the most vulnerable families living with an OVC in Ziway; the availability of registration or acknowledgment as the parent or PCG in the FHE Ziway Child Development Program database; the OVC having been supported by FHE for at least the last three years; the documentation being available for the OVC’s initial personal case history in the FH Archives; the OVC’s and PCG’s residence in the Ziway township or area for at least the last three years and speaking the local or national language; the OVC and PCG having been visited by a Food for the Hungry community-level worker or volunteers over the previous three years; and lastly, living with an OVC over ten years of age, with FHE having supported the child for at least three years, and attending school.

Six research respondents were drawn from OVCs’ PCGs within the FHE child development program in Ziway. In the program’s context, the PCGs of orphaned children are usually single parents, grandparents, biological family members, or guardians with no blood relationship. These families are identified as the poorest of the poor in the community. These 6 cases are referred to throughout this paper as C-1 (Case 1) through C-6.

### 2.5. Data Analysis

This study applied a logic-based thematic analysis to triangulate information from all the source data gathered. ATLAS.ti 23 was used for open coding and categorizing specific themes from case interviews and informant group discussions. Logical and analytical methods were also applied to create a meaningful link across the findings under each theme pooled from different sources. This approach was initially formulated in 1979 by Joseph Wholey [42]. He used a logical method to assess the outcome/result through a cause–effect connection between the output (intermediate outcome) and the outcome. Robert Yin also suggested using a logical technique for case study research analysis in integrating observed events and repeated cause–effect linkages into theoretical prediction [43]. The focus group discussion and individual interviews were carried out with 11 key informant community promoters (para-social workers) assigned to assist and follow up on the OVCs’ and caregivers’ well-being conditions. Informal individual interviews were also conducted with the program director, field operation manager, and four community properties closely working with the OVCs and their caregivers over the previous three years. The individual informal interviews allowed some interviewees to feel greater confidence and freedom to discuss sensitive topics that could be unlikely in larger formal group discussion settings. Seven semi-structured questionnaires were used for key informant focus group discussion and informal individual interviews, corresponding with seven major themes. The themes were caregivers’ burden, a sense of joy, caring and nurturing roles, friends, supportive community, worries for the future, and ways to improve their well-being and quality of life. The analysis of this study used a reduced form of the conceptual framework and reported findings on the assessment of PCGs’ well-being using a WMT (36-item scale), perceived stress linked with PCG burden and OVC caring roles, and potential supportive mechanisms PCGs utilized as coping means.

## 3. Results

### 3.1. Demographic Description

The caregivers resided in three localities (villages). Except for one case (C-2), the rest of the OVCs’ PCGs were female, and their ages ranged between 46 and 67. Two cases (C-6 and C-8) had large family sizes of six and eight, respectively, C-1 had five (average), and the rest had a small family size. C-3, -4, and -6 had the highest number of children (four each), whereas C-3 had only one child. PCGs’ religious affiliations were described as Muslim (C-1 and -4), Orthodox (C-5 and C-6), and Protestant (C-3). Their relationships with the OVCs were indicated as grandparents (C-1, C-3, and C-5), biological parents (C-2 and C-6), and non-blood-relationship guardians (C-4). All of them led their lives with unstable means of income and did not have a constant source of monthly income as they faced health-related challenges and were limited by other environmental and seasonal weather conditions, such as extreme heat, rainy seasons, and unavailability of work. The participants had no formal and elementary-level education, and their livelihood was based on selling small goods in the open local market, daily-type labor work, and the monthly minimal support provided through FHE.

According to the Oromia Region Socio-Economic profile report (2007), Orthodox Christianity is the dominant religion (51.04%), followed by Islam (24.69%) and Protestantism (22.07%), leading to occasional religious tensions [44]. The livelihoods of the population in the Ziway area depend on subsistence agriculture and seasonal rainfall, with only 21.6% of land cultivable, while 15.7% is swampy and 25.6% degraded. Fishing and labor in the flower farming industry, such as the Dutch company Sher, which employs over 1500 workers [45], provide alternative income sources. However, ecological pollution has become a significant concern, as flower farms around Lake Ziway have damaged the ecosystem, affecting the livelihoods of thousands of fishers and locals [46]. These socioeconomic and environmental challenges make it difficult to achieve economic stability and provide proper childcare, especially for uneducated orphaned and vulnerable children (OVCs) and primary caregivers (PCGs) facing significant well-being deficiencies [44].

### 3.2. OVCs’ Caregivers Well-Being Assessment

**Figure 1:**
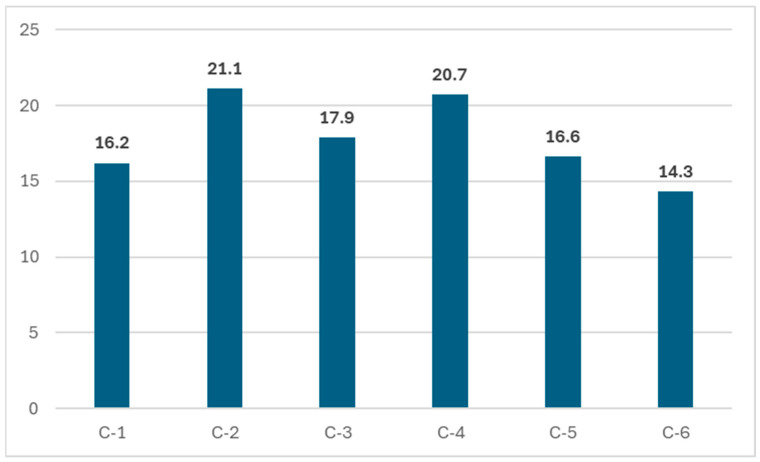
Well-being domain summary mean scores.

**Figure 2:**
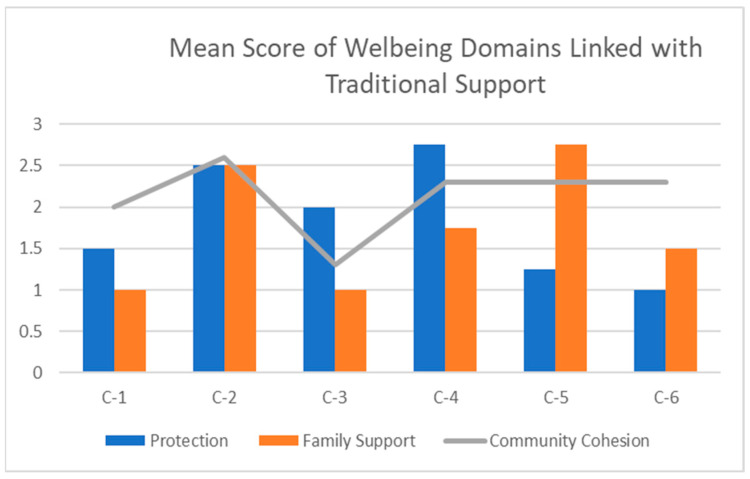
Traditional support domains.

Figure 1 and Figure 2 summarize OVCs’ PCGs’ well-being conditions per domain and individual cases. A WMT with a 36-item scale was used to measure ten domains of well-being. Each domain was assessed with three to four questions, and the summative score ranges of the ten domains were between 10 and 30. For a single well-being domain, the desired, average, and deficiency scores are 2.5, 2.3, and 1.5, respectively. The scale was widely used in low-income SSA regions, including Ethiopia, Kenya, and Rwanda, for individual monitoring and macro well-being impact assessment targeting OVCs and their households [37]. The scale encompasses ten major well-being domains: food and nutrition, shelter, economy, general health, education, mental health, protection, family support, spirituality, and community cohesion. To assess the conditions of traditional and community support, participants were asked to rate the level of support they received using several guiding questions, such as “At home, I have someone to look after me if I get hurt, sick, or feel sad”, “I receive the emotional support and help I need from my family”, “I have people I can trust”, “I receive free support to care for the children who live with me”, etc. The desirable and average summative well-being domain scores were 25 and 23, respectively. A score of less than 23 indicated PCGs’ experience of some deficiencies within the domain. A score below 15 indicated critical deficiencies within the domains requiring immediate intervention. Accordingly, as indicated in Figure 1, none of the PCGs had desirable or average well-being domain scores. As illustrated in Figure 1, the summary average well-being scores for the six cases were 16.2, 21.1, 17.95, 20.7, 16.55, and 14.25, respectively, for Cases 1 through 6. Instead, they were all experiencing deficits, and C-6 showed a critical emergency well-being condition.

### 3.3. OVCs’ PCGs’ Perceived Stress

Caregiving for OVCs has a mental, physical, social, psychological, and spiritual impact, and its negative effect worsens where poverty prevails and is among the most vulnerable primary caregivers [47]. PCGs’ stress relates to the holistic developmental needs of their OVCs and their well-being conditions, especially the need for basic needs, health, and financial limitations. PCGs under the FH Integrated Community Development Program (ICDP) were identified and qualified as “the most vulnerable household” living with one or more school-age children. Most OVCs live with their grandparents, who have some well-being deficiencies such as chronic physical and mental illnesses and extreme socioeconomic limitations. In Ethiopia, most PCGs base their means of living on daily labor work, selling small goods such as cooking charcoal and vegetables in the local market, or they are dependent on family members or institutional support. The economic limitations and social, physical, and psychological challenges in addressing their children and their needs leave them in a stressful situation affecting their well-being [4]. Unmet basic child needs and caregivers’ mental, psychological, and emotional conditions also affect a child’s behavior and parental attachment daily [4].

The in-depth qualitative investigation also supported the general assessment of PCG well-being findings explored through the WMT survey. In line with the study aims, participants were asked open-ended questions, such as “What can you say or feel about being a caregiver?”, “How do you perceive the level of your burden?”, “During times of need, who or which group of people do you think is helping you?”, “What are the sources of your burden?”, “Do you have close friends or others who visit you and your child and have tried to help you during times of need?”, etc. All caregivers expressed their perceived stress in multiple areas of well-being domains, and some with deep emotion (crying) and feelings of hopelessness. For example, the monthly organizational (FHE) in-kind support was the main survival means that C-1, C-2, C-3, and C-4 were dependent on, providing 15 kg of wheat flour, ½ liter of cooking oil, 1⁄2 Kg of beans, and ETB 300 (USD 13.6). An HIV-infected grandmother of a 12-year-old OVC living with HIV-AIDS said, “God also gave us a few friends from the church who always welcome us; we don’t worry about what to eat for the holidays”. A 46-year-old and mother of six children said,

“We sold our land, the only property to feed our children. Now, nothing is left except me, and my four children are dying in an empty house. God gave me these twins; they are 11 months old but still not properly sitting. My husband left the area, and I can’t throw them and disappear. My son is in grade 3 but 11, but he is not attending school properly as he is helping me take care of his siblings. (4 by 4-m mud house), two of my older children (13 & 15 years) left and work for somebody else”.

### 3.4. OVCs’ PCGs’ Caring Roles and Traditional Family and Community Support

Ethiopia’s strong relational and support culture intervenes in the family (nuclear and extended family) and neighborhood connectedness. The famous African proverb can illustrate these extended family roles in the parenting system and nurturing home, “it takes a village to raise a child” [48]. Childcare and nurturing are both an art and a culturally acquired behavior, skill, value, and practice, as individuals learn from their parents’ and families’ parenting. Childcare in Ethiopian culture is not confined to biological parents; extended family and trusted neighbors also share the caring role. In Ethiopia’s traditional culture, children are consistently encouraged to develop a strong sense of family and social responsibility early in childhood, and grandparents and older people play a role in imparting family values and other collective norms and values [49]. However, due to a paradigm shift, traditional African parenting is no longer the same and is becoming fragile, as postmodern values are growing. Poverty, unemployment, population increases, and urbanization are the major factors affecting traditional African parenting as young people leave their families and extended families [18]. However, the African saying “a single hand cannot nurture a child” is still strong in the rural Ethiopian traditional parenting and child-nurturing culture. But parental loss, poverty, migration, and the immigration of young parents are shifting this rupturing role to elderly grandparents, while the intervention of humanitarian organizations is also required. This study assessed OVCs’ PCGs’ social support conditions and experiences by three well-being domain conditions: protection, family support, and community cohesion. Findings from the in-depth caregiver interviews and key informant group discussions were triangulated to enrich and validate the qualitative results.

### 3.5. Perceived Stress from Protection Problems

The protection domain assessed whether the PCGs were treated differently from others in the community, school, and households. The results showed that only C-2 and C-4 reported the desirable protection condition (score equal to or above 2.5), while C-1, C-5, and C-6 indicated the lowest score, which indicated deficiencies in protection (i.e., perceived as unprotected). Data from the case interview revealed that for C-1 and C-5, the perceived stress regarding protection was likely connected with their OVCs’ health condition, particularly HIV-AIDS. They also perceived that they were not treated in the same way that other people were treated in the neighborhood and by other community groups, such as during community meeting times. These PCGs reported being discriminated against because they were connected with HIV. C-1, a 67-year-old grandmother caring for three OVCs, said:

“Before HIV took my only daughter, people in these villages were my customers and bought what I was selling in the market. But after she died, my two grandsons and I were lonely and isolated. It seems that even our neighbors are afraid to get closer to us. These days I am selling only cooking charcoal, and I am not going to the marketplace as I used to; I am sick, I have cancer and have undergone radiation 12 times”.

Similarly, C-5, a grandmother who was caring for eight OVCs, said,

“My life is full of fear! I lost two of my daughters and my son-in-law because of HIV. Three of my grandchildren also live with the virus, and the older one refused to take medicine (ART), and she is at Ziway Hospital; she prefers ding. She attempted suicide twice after losing her parents and knew she was infected with this “satanic disease”. Slowly my neighbors and friends are getting distant and have stopped coming. I am not judging them that the common reaction; we lived together and had good times. I could have died before all this despair; what is left for me?”.

Nonetheless, C-6 perceived the strongest protection defect (crisis); her story was exceptional and unconnected with HIV-AIDS or other health-related issues. She and her family were living in fear of harm. She and her household perceived and experienced rejection, hate speech, discrimination, and verbal abuse. According to C-2:

“We are strangers (from the North region), and don’t speak the local language “Oromigna”. In these areas, there is ethnic tension; they hate people from the North; people were killed recently”. “We don’t belong here, but we don’t have any option”. “These days, I am afraid to send my children even to the nearby kiosks. My son was bitten twice in school; now he hates attending school”.

## 4. Family Support

The family support well-being domain measured whether the PCG perceived that their family members and relatives supported them. As indicated in Table 1 and Figure 2, caregivers’ perceived family support was assessed by the WMT. Participants were asked about the support they received from their family members and relatives using structured and semi-structured questionnaires. The WMT score showed a desirable family support score for C-2 and C-5 but deficiencies for C-1 and C-3. During the interview, C-2, a 49-year-old sick father raising an 11-year-old boy, mentioned that, after the death of his wife, his sister and her husband visited and supported them frequently.

For C-5, the ownership of assets allowed her to maintain some family members and receive material physiological support. In this study, C-5 was the only PCG with few assets, such as a house, farming tools, and land. Some of her family members used her farming tools, worked on her land, and shared the “corn and cabbages” production with her. On the contrary, C-1 and C-5 indicated deficiencies in family support connected with AIDS and being perceived as a stranger in the area (being away from family connection), respectively. C-6 said, “We left our family and relatives a long time ago; I don’t know where they are at the age of our second child (13 years ago). We couldn’t travel back and see them; it is too far, and the transportation is expensive. Relatives are like your shield; in this area, I feel like we are living “koliyachienine” just like a necked person”. C-1 said, “I am not from this area, but my husband. But he died ten years back, and my daughter and his relatives don’t want to see the grandkids. Even before, we didn’t have that much connection because of my husband; he was jealous and didn’t want me to connect with relatives and other people”.

### Community Cohesion

The community cohesion well-being domain and structured and non-structured questions mainly assessed and explored the feelings of PCGs on the condition of being welcomed in the community. As indicated in Table 1 and Figure 2, except for C-2, the rest reported more critical deficiencies. They indicated that they were neither receiving support nor being welcomed by the community they were living in. However, C-6 felt her children were accepted and receiving help from her neighbor, who was also initially from another region. C-6 said,

“I only go to the marketplace when neighbors are around and look after my 11-month twins. Sometimes I send her to different places, like my younger sister or daughter. People from this area are very cruel; not compassionate at all. They looked at me and told me my children would not survive”. “I am not going anywhere unless my neighbor is available for them; it is hard to trust others”.

Except for C-3, all the rest perceived that the religious community welcomed them. In contrast, C-2 perceived a strong connection with neighbor and religious affiliation. He was so thankful to his neighbor and said,

“After his wife passed away, my neighbor was always available for my son to feed him after school and take him to the clinic whenever he was sick. She kept the promise she made to me to my wife (her good friend) while she was on the verge of death to take care of my son. She is like a mother to him. Allah doesn’t quarrel (took my wife and kept me sick) without providing the way out to my son”.

## 5. Discussion

The results of this study shed light on the theoretical assumptions regarding changes in social and family dynamics in African parenting and interpersonal relationships. Despite the growing humanitarian challenges, the shift in primary child caregiving from biological parents to extended families, grandparents, and guardians—often involving friends and neighbors—shaped both the framework and the findings of this study. As the results and previous studies indicate, the complex outcomes of poverty, HIV, and poor well-being conditions are among the leading contributing factors. These factors have exacerbated the disintegration of traditional structures and created a more sensitive social environment, impacting family values. As individuals, groups, families, and communities strive for independent survival and stable well-being, the availability and willingness to extend support has diminished over time. Additionally, the low socioeconomic conditions and demographic characteristics of participants, along with the well-being deficiencies observed in their livelihoods (C1–C6), particularly in those who reported critical deficits and perceived stress, such as C6, serve as a clear indicator of these challenges, and require evidence-based solutions.

Studies in SSA and other low-income countries have indicated an increase in the OVC population and the need and urgency to address their developmental needs and provide care and support for vulnerable PCGs [9,10,18]. Poverty, war, and prolonged human crisis have left millions of children without parents and are shifting the role of OVC care to grandparents in Ethiopia. The socioeconomic limitations and physical disabilities of OVCs’ PCGs and the challenge of addressing the basic needs of their OVCs are linked with their key well-being deficiencies and perceived stress [13,14,22]. Nonetheless, Ethiopia has no centralized or organized local community system assisting PCGs and their OVCs’ livelihood. In this study, perceived stresses were explained as physical disabilities; extreme socioeconomic challenges including lack of stable income and shelter; emotional stress from the loss of children, grandchildren, and HIV-infected OVCs; and hopelessness linked with worries for the future survival of their OVCs. The average summative mean scores from ten well-being domains indicated that the PGCs were experiencing well-being deficiencies and crises. More specifically, the analysis of the results from the protection well-being domain indicated that, while C-2 and C-4 reported a desirable protection score, C-1, C-5, and C-6 indicated they felt unprotected and feared hate and harm from others, respectively. In the SSA social system, family and community connections are strong elements in providing care and support for needy family members and neighbors [30]. However, African families and extended families’ values and social support heritages are being eroded and challenged by the postmodern ideologies and demands of global changes and challenges, which likely result from increases in unemployment, socioeconomic deprivation, inflation and standard-of-living inflation, internal and external migration, poverty, and other resource-based tensions and conflicts. The PCGs in this study perceived a lack of visits, communication, and other supportive treatment from their family members, neighbors, and community. Similarly, a report from the well-being survey showed that C-1, C-3, C-4, and C-5 (scores below the average) indicated deficiencies in the family domain that assessed connectedness and care from family members. Further, C-1 and C-3 reported defects in the community cohesion well-being domain, which assessed their feeling of being accepted and welcomed by their close community.

## 6. Limitations

Despite this study’s contribution to the evidence regarding OVCs’ PCGs’ well-being conditions, stress, and the role of family and community support, the study has limitations: First, the findings are based on a case study exploration, and do not establish generalizability and causality. Second, a few cases were selected based on the criterion of purposive sampling that does not represent other cases in the program; it lacks a representative sample. However, the findings could contribute to further comprehensive investigations, intervention science, and practices that address well-being issues in a low socioeconomic environment.

## 7. Conclusions

This study highlights the increasing burden on primary caregivers in Ethiopia, as caregiving shifts from biological parents to extended families, compounded by severe socioeconomic challenges. The weakening of family and community support systems—driven by poverty, HIV/AIDS, migration, and unemployment—reveals the urgent need to rebuild these networks to support vulnerable caregivers and orphans and vulnerable children (OVCs). Additionally, this study connects perceived stress to significant well-being deficiencies in caregivers, caused by factors such as a lack of stable income, emotional strain, and physical limitations. These findings emphasize the need for targeted, evidence-based interventions that offer financial, emotional, and community support to improve outcomes for both caregivers and children. Caring and nurturing for OVCs is a demanding role in a complex poverty context. Disabilities and socioeconomic limitations left millions of OVCs’ PCGs in Ethiopia with well-being deficiencies, overwhelming burdens, and being overlooked, unwelcomed, and in a silent emergency. Various social limitations, including a lack of proper family support and community cohesion, could be sources of stress for OVCs’ PCGs in Ethiopia, particularly for the older, disabled, and vulnerable. Close monitoring should be provided to HIV patients and large-family-size vulnerable PCGs to monitor the source of the well-being stressors threatening their lives. Family and community support is not strong in extending care and support, and most OVCs’ PCGs are left to the support of humanitarian aid. A lack of family and community support could worsen feelings of being overlooked, unwelcome, and lonely. There is a need for a centralized sustainable community and other humanitarian organizations’ care and support systems that could help OVCs and their PCGs to be self-reliant and improve their and their OVCs’ living situation and well-being conditions. This study recommends further rigorous and comprehensive scientific research examining the well-being determinants of OVCs and their PCGs in Ethiopia and the role of support systems in both rural and urban contexts. A policy that promotes a supportive community environment and safeguards the most vulnerable OVCs and PCGs should be in place.

## Figures and Tables

**Table 1:** Mean scores for traditional support well-being domains.

List of Cases	Protection	Family Support	Community Cohesion
C-1	1.5	1	2
C-2	2.5	2.5	2.6
C-3	2	1	1.3
C-4	2.75	1.75	2.3
C-5	1.25	2.75	2.3
C-6	1	1.5	2.3

## Data Availability

The data presented in this study are available on request from the corresponding author due to privacy concerns.
